# Preexisting Antibodies to an F(ab′)_2_ Antibody Therapeutic and Novel Method for Immunogenicity Assessment

**DOI:** 10.1155/2016/2921758

**Published:** 2016-06-16

**Authors:** Jane Ruppel, Ann Brady, Rebecca Elliott, Cecilia Leddy, Marco Palencia, Daniel Coleman, Jessica A. Couch, Eric Wakshull

**Affiliations:** ^1^BioAnalytical Sciences Department, Genentech, 1 DNA Way, South San Francisco, CA 94080, USA; ^2^Biosample and Repository Management Department, Genentech, 1 DNA Way, South San Francisco, CA 94080, USA; ^3^Nonclinical Biostatistics Department, Genentech, 1 DNA Way, South San Francisco, CA 94080, USA; ^4^Safety Assessment Department, Genentech, 1 DNA Way, South San Francisco, CA 94080, USA

## Abstract

Anti-therapeutic antibodies (ATAs) may impact drug exposure and activity and induce immune complex mediated toxicity; therefore the accurate measurement of ATA is important for the analysis of drug safety and efficacy. Preexisting ATAs to the hinge region of anti-Delta like ligand 4 (anti-DLL4) F(ab′)_2_, a potential antitumor therapeutic, were detected in cynomolgus monkey serum, which presented a challenge in developing assays for detecting treatment induced ATA. A total ATA assay was developed using a bridging ELISA that detected both anti-CDR and anti-framework ATA including anti-hinge reactivity. A competition assay that could detect 500 ng/mL of anti-CDR ATA in the presence of preexisting ATA was also developed to determine ATA specific to the anti-DLL4 F(ab′)_2_ CDR using anti-DLL4 F(ab′)_2_ and a control F(ab′)_2_. We used these assay methods in a cynomolgus monkey in vivo study to successfully evaluate total and anti-CDR ATA. The preexisting anti-hinge reactivity was also observed to a lesser extent in human serum, and a similar approach could be applied for specific immunogenicity assessment in clinical trials.

## 1. Introduction

The administration of large molecule protein drugs can result in the development of antibodies against the therapeutic protein, which may lead to loss of efficacy [1] and alteration of clearance or induction of immune-mediated toxicities. Assessment of these anti-therapeutic antibodies (ATAs) responses is important for interpretation of relevant endpoints including pharmacokinetics, pharmacodynamics, safety, and/or efficacy of the molecule [2–4]. ATA can affect drug responses by decreasing drug exposure through clearance of large protein : ATA complexes [5]. Conversely, clearance can be decreased for proteins that are contained in immune complexes, leading to accumulation of total protein [6]. In addition, an Fab or F(ab′)_2_ antibody that does not itself have effector functions such as antibody-dependent cell-mediated cytotoxicity (ADCC) may have this function reconstituted through the Fc portion of ATA that is present in the drug : ATA complex [7]. These have potential safety implications such as induction of immune-complex toxicities such as vasculitis. Finally, drug activity may be neutralized by anti-complementarity determining region (anti-CDR) ATA reactivity that blocks the bind of drug to its target [8].

Delta like ligand 4 (DLL4) is a member of the Notch signaling pathway [9, 10]. DLL4 inhibition impairs tumor growth by disrupting the balance of tip and stalk cells of sprouting endothelium and thus promoting nonproductive angiogenesis [11, 12]. Although anti-DLL4 full length antibody showed potent antitumor activity, nonclinical in vivo testing resulted in unmanageable toxicity, with vascular and liver toxicities [13]. Therefore a rapidly cleared F(ab′)_2_ form of a humanized anti-DLL4 monoclonal antibody was generated to ameliorate toxicity while maintaining efficacy [14] by reducing drug exposure but maintaining sufficient target engagement. During development of an ATA assay for this F(ab′)_2_ molecule, we observed a high prevalence of preexisting reactivity to anti-DLL4 F(ab′)_2_ in cynomolgus monkey serum samples from drug naïve animals. These sera did not react with the precursor full length antibody molecule but did react with both the anti-DLL4 F(ab′)_2_ and an F(ab′)_2_ prepared from a different humanized monoclonal antibody with the same framework residues but with a different CDR sequence. This indicates that the preexisting reactivity is directed to the hinge epitope that is exposed when the Fc fragment is enzymatically removed to generate the F(ab′)_2_ from the full length antibody. It has been reported that anti-hinge antibody reactivity can be highly specific to the exact IgG cleavage site [15, 16]. The observed cross reactivity of the cynomolgus monkey preexisting anti-hinge antibodies to the human F(ab′)_2_ molecule suggests that there is a high degree of homology between the human and cynomolgus monkey hinge epitopes.

Fab and F(ab′)_2_ fragments are known to be generated in vivo by certain bacterial proteases, probably as a survival mechanism by preventing anti-bacterial antibodies from utilizing effector activities [17]. Anti-hinge antibodies have been reported by other researchers and have been linked to various in vitro and in vivo effects including reconstitution of effector activity [7, 18, 19]. In a study of several therapeutic drugs where F(ab′)_2_ fragments were used to avoid rheumatoid factor interference, an increase in ATA assay background was observed due to anti-hinge IgG in human serum reacting with drug F(ab′)_2_ [20]. A therapeutic F(ab′)_2_ anti-glycoprotein IIb/IIIa drug intended to prevent platelet aggregation unexpectedly resulted in a decrease in platelets in treated cynomolgus monkeys, probably due to reconstitution of Fc effector function by anti-hinge antibodies [21]. Higher preexisting anti-hinge antibody activity has also been correlated with kidney transplant survival [22]. Stimulation of complement activation by complexes of anti-hinge antibodies with F(ab′)_2_ has also been reported [23].

In this study, we describe methods to evaluate both ATA to the entire F(ab′)_2_ molecule and to also evaluate anti-CDR ATA. Use of these methods can potentially enable interpretation and analysis of various mechanistic effects due to ATA development.

## 2. Materials and Methods

### 2.1. Materials

Anti-DLL4 F(ab′)_2_ was prepared by pepsin cleavage as described in Couch et al. [14]. Anti-DLL4 Fab was prepared by standard papain digestion methods at Genentech. Herceptin® F(ab′)_2_ was prepared by standard pepsin digestion methods at Genentech. Affinity purified anti-CDR antibody to anti-DLL4 F(ab′)_2_ was prepared by immunizing goats with recombinant human anti-DLL4 antibody Fab fragments on days 0, 14, 28, 42, and 56, followed by serum collection on day 66. The immunized goat antiserum was affinity-enriched for anti-CDR antibodies using immobilized full length anti-DLL4 coupled via primary amines to an agarose column followed by elution with 0.1 M glycine pH 2.5. Remaining anti-framework antibody was removed by adsorbing the eluate over a column coupled with a framework control antibody. Cynomolgus monkey serum samples from untreated animals were purchased from BioreclamationIVT (Hicksville, NY) and Covance (Westbury, NY). Detection conjugate for the PK assay was prepared using 10C4, a mouse monoclonal antibody that recognizes anti-DLL4 in the presence of human IgG [24], coupled to horseradish peroxidase (HRP) as described by the manufacturer (Pierce Plus Activated Peroxidase, ThermoFisher Scientific, Waltham, MA).

### 2.2. Direct ATA Assay

Anti-DLL4 F(ab′)_2_ diluted to 1 *μ*g/mL in carbonate buffer pH 9.6 was added to a high binding polystyrene ELISA plate (Nunc ThermoFisher, Waltham, MA) and incubated overnight at 4°C. The plate was washed with wash buffer (PBS/0.05% polysorbate 20) and remaining binding sites were blocked using assay diluent (PBS, 0.5% BSA, 0.05% polysorbate 20, 0.05% ProClin 300, pH 7.4).

After incubation and washing, samples diluted in assay diluent were added and incubated for two hours. The plate was washed, and captured ATA was detected by adding donkey anti-human IgG Fc-specific HRP conjugate (Jackson, West Grove, PA). After incubation and washing, signal was generated by adding tetramethylbenzidine (TMB; Moss, Pasadena, MD) and stopping the reaction with phosphoric acid. Absorbance was measured at 450 nm using 650 nm reference (Molecular Devices, Sunnyvale, CA).

### 2.3. Bridging ATA Assay

Anti-DLL4 F(ab′)_2_ was conjugated to biotin using sulfo-NHS-LC-biotin (Pierce/ThermoFisher, Waltham, MA) or digoxigenin using 3-amino-3-deoxydigoxigenin hemisuccinamide succinimidyl ester (Invitrogen, Carlsbad, CA). Master mix was prepared as a mixture of both conjugates, each at 2 *μ*g/mL, in assay buffer. Samples were diluted 1/20 in assay diluent and then titered with seven subsequent 1/5 dilutions. Equal volumes of master mix and diluted sample were mixed and incubated overnight.

This reaction mixture was then incubated in a washed high binding streptavidin plate (Roche, Indianapolis, IN). After washing, bound antibody : conjugate complexes were detected by adding mouse monoclonal anti-DIG HRP conjugate (Jackson ImmunoResearch, West Grove, PA) to the streptavidin plate. After washing, signal was generated by adding TMB (Kirkegaard & Perry, Gaithersburg, MD), stopping the reaction with 1 M phosphoric acid. Absorbance was measured at 450 nm using a reference wavelength of 630 nm using an Infinite 200 spectrophotometer (Tecan, Switzerland).

Goat anti-DLL4 F(ab′)_2_ CDR purified antibody was used as a positive control. Assay buffer was used as a negative control due to the preexisting anti-F(ab′)_2_ antibodies observed in cynomolgus monkey serum. In the final bridging assay, concentrations as low as 500 ng/mL of positive control antibody could be detected in the presence of up to 2 *μ*g/mL anti-DLL4 F(ab′)_2_.

### 2.4. Bridging ATA Assay Using Competitive Molecules

Molecules for competition were prepared at 100 *μ*g/mL in assay diluent. Cynomolgus monkey serum samples were first diluted 1/10 into assay diluent and then mixed in a 1 : 1 ratio with either diluted competition molecule solution or assay diluent, resulting in a final sample dilution of 1/20 serum with 50 *μ*g/mL final competitor concentration. The mixture was incubated with agitation for at least one hour at room temperature to allow complex formation before addition of the Master Mix. These dilutions were assayed using the bridging ATA assay format.

### 2.5. PK Assay

Recombinant human DLL4 extracellular domain diluted to 1 *μ*g/mL in pH 9.6 carbonate buffer was added to a high binding polystyrene ELISA plate (Nunc ThermoScientific, Waltham, MA) and incubated overnight at 4°C. The plate was washed and assay buffer was added to block any remaining binding sites.

After incubation and washing, the diluted serum sample was added to capture anti-DLL4 F(ab′)_2_ in the sample.

The plate was washed, and captured anti-DLL4 F(ab′)_2_ was detected by adding 10C4-HRP conjugate, a mouse monoclonal antibody that recognizes anti-DLL4 in the presence of human IgG [24] coupled to HRP. After incubation and washing, signal was generated by adding TMB (KPL), stopping the reaction with phosphoric acid. Absorbance was measured at 450 nm using 620 nm reference wavelength on a Tecan Infinite ELISA plate reader (Tecan).

## 3. Results and Discussion

During development of an ATA assay for a full length therapeutic antibody, assay signals from untreated animal sera are generally used to set the ATA positive threshold (cutpoint), which differentiates ATA negative and positive samples, by calculating the signal variance using a target 5% false positive ATA rate [25]. Because we observed that almost all cynomolgus monkey serum samples from treatment-naïve animals gave high and variable responses during the development of the anti-DLL4 F(ab′)_2_ ATA assay, this approach could not be used. Therefore, we developed a new approach based on setting individual cutpoints for each animal. First we determined that the preexisting reactivity was specific to the neo-epitope at the F(ab′)_2_ hinge region. We then evaluated the expected temporal changes in this reactivity in the absence of any drug treatment. We developed robust methods using individual cutpoints for determining both changes in titer to the entire molecule and methods for assessing changes in ATA binding epitopes on anti-DLL4 (i.e., hinge neo-epitope versus anti-CDR specific ATA).

### 3.1. Preexisting Reactivity Is Specific for Anti-Hinge of F(ab′)_2_ but Not for Full Length Molecule as Shown by Competition

**Figure 1:**
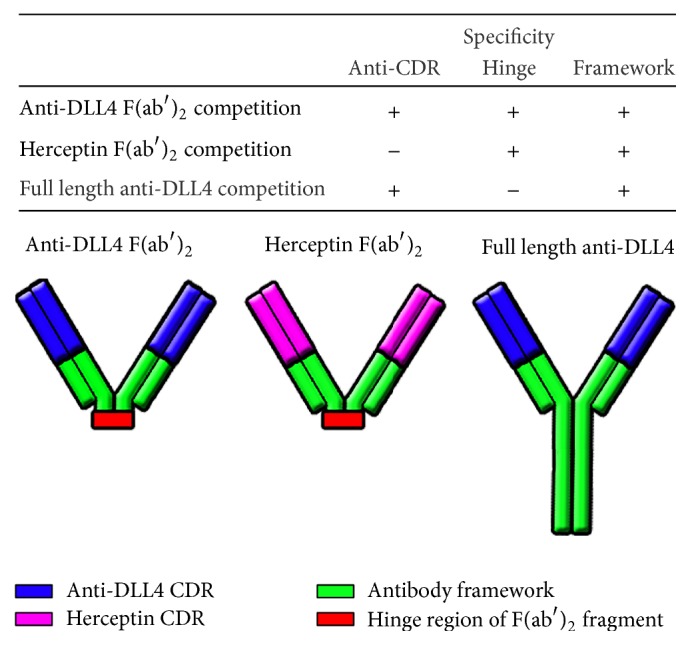
Specificity of signal reduction in the anti-DLL4 F(ab′)_2_ ATA assay by different competitor molecules. Anti-DLL4 F(ab′)_2_ competitor will reduce all reactivity in the ATA assay. Control F(ab′)_2_ will not reduce anti-DLL4 F(ab′)_2_ CDR reactivity but will reduce F(ab′)_2_ framework and hinge reactivity. Anti-DLL4 full length antibody will reduce anti-DLL4 CDR and framework reactivity, but not hinge reactivity.

**Figure 2:**
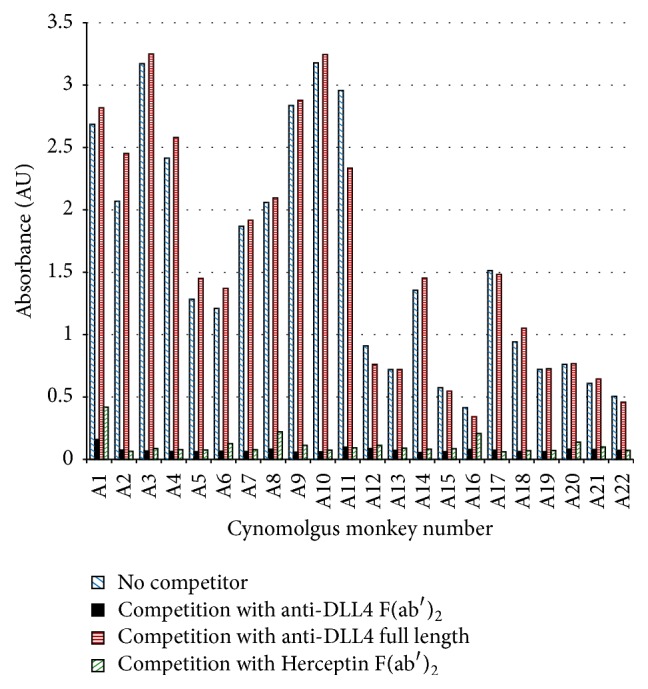
Preexisting ATAs are directed primarily at the F(ab′)_2_ hinge, as shown by the reactivity reduction by competition with anti-DLL4 F(ab′)_2_, Herceptin F(ab′)_2_, or anti-DLL4 full length antibody in the bridging ELISA format. In the bridging ATA assay using 50 *μ*g/mL competitor (in-well concentration) and a 1/20 sample dilution, full length antibody causes very little signal reduction; however the F(ab′)_2_ form of the same antibody reduces the signal almost to background. An F(ab′)_2_ form of Herceptin, which has the same framework as anti-DLL4 but with a different CDR, also reduces the signal, indicating that the signal is due to anti-hinge antibodies.

The specificity of the preexisting ATA antibodies found in cynomolgus monkey serum was mapped using the strategy shown in Figure 1. Serum samples were tested using a competition assay that contained either no competitor, anti-DLL4 F(ab′)_2_, or full length anti-DLL4. In most samples, the reactivity was reduced by anti-DLL4 F(ab′)_2_ but not by the full length antibody, indicating anti-hinge specificity. Binding of cynomolgus monkey ATA in the bridging ATA assay is blocked by F(ab′)_2_ fragments from anti-DLL4 and from another control monoclonal antibody but is not blocked by anti-DLL4 full length antibody, indicating that the reactivity is specific for the hinge region of anti-DLL4 F(ab′)_2_ (Figure 2).

### 3.2. Preexisting Reactivity Is Specific for the Hinge Neo-Epitope on the F(ab′)_2_, Since Little Reactivity Is Seen for Fab as Shown by Direct Binding Assay

**Figure 3:**
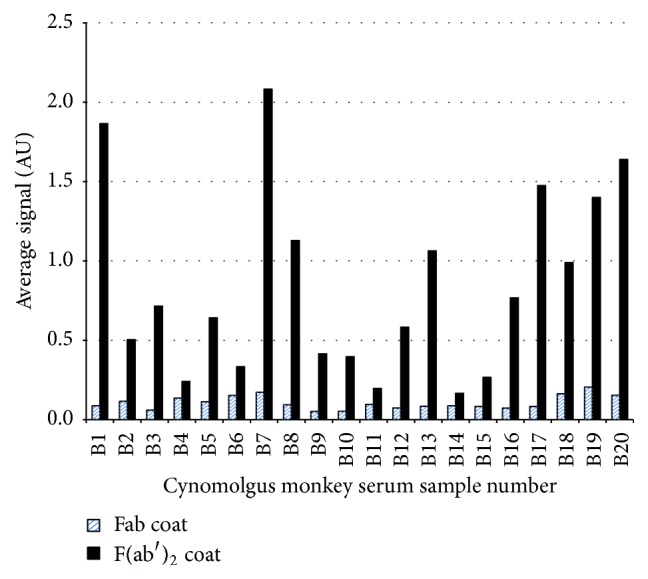
Comparative reactivity of cynomolgus monkey serum samples with anti-DLL4 Fab or anti-DLL4 F(ab′)_2_ directly coated on a well (direct binding format). More sample reactivity is observed with the F(ab′)_2_ coat.

Direct detection of preexisting anti-hinge IgG in a panel of 20 individual cynomolgus monkey serum samples was tested using anti-DLL4 F(ab′)_2_ or anti-DLL4 Fab coated onto ELISA wells, with detection using anti-human IgG Fc specific HRP labeled conjugate. Papain-generated Fab fragment is 10 amino acids shorter in the hinge region than pepsin-generated F(ab′)_2_ fragment and is monomeric rather than dimeric [17]. Sample reactivity to the F(ab′)_2_ coat was readily observed while little reactivity was seen when plates were coated with the Fab fragment, thus confirming reactivity against the hinge epitope on the F(ab′)_2_ fragment (Figure 3).

### 3.3. Preexisting Reactivity Is Stable within Individual Animals over 14 Days but Varies Considerably between Animals

**Figure 4:**
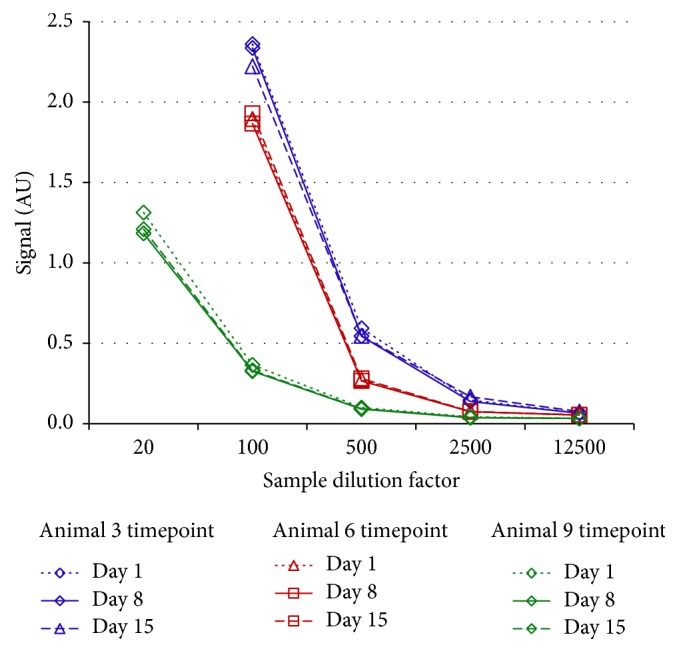
Representative anti-DLL4 F(ab′)_2_ bridging ATA assay titer curves from three cynomolgus monkeys sampled weekly, three samples per animal total. The signals were consistent within animal but varied widely between animals.

In order to determine whether the overall reactivity to the drug is changed after the animal is treated, a qualitative assessment of the temporal response variability within an animal without any drug treatment was done. In this way the magnitude of an induced change in assay signal could be distinguished from longitudinal variation in the preexisting signal. A series of three samples was collected from each of ten treatment-naïve cynomolgus monkeys over 14 days and the samples were titered in the bridging ATA assay. Overall consistent titers were observed within animal week to week, but in contrast the titers varied widely between animals at the same time points. Representative data are shown below. Thus, preexisting reactivity is stable within individual animals over 14 days but varies considerably between animals (Figure 4).

In this study we used the two pretreatment samples from each animal for individual cutpoint calculation, but the use of the control group variation in antibody titers over the course of the study to define the pooled standard deviation could also be used to refine the estimated antibody temporal variation that is unrelated to treatment.

### 3.4. Anti-CDR Can Be Detected in the Presence of Preexisting Reactivity Using Competition with Full Length Antibody or F(ab′)_2_, but Competition with F(ab′)_2_ Has Better Dynamic Range

Given the robust preexisting ATA responses seen in cynomolgus monkey samples, the challenge was confirming a small anti-DLL4-specific anti-CDR signal on top of a large anti-DLL4-nonspecific assay signal generated by preexisting anti-hinge ATA. Two assay formats were explored for the detection of anti-CDR antibody in the presence of high levels of preexisting anti-hinge antibodies. We evaluated these formats for their ability to detect a low concentration of anti-CDR antibodies in the presence of high levels of anti-hinge antibody. We found that anti-CDR can be detected in the presence of preexisting reactivity using competition with full length or F(ab′)_2_, and that competition with F(ab′)_2_ gave assay signals with a better dynamic range.

We used a competitive molecule concentration of 50 *μ*g/mL in a 1/20 sample dilution. This concentration was shown during assay development to reduce most of the signal in samples with preexisting reactivity.

The first assay format used competition with full length anti-DLL4 in the bridging assay. Anti-CDR antibodies are detected by calculating the change in signal between sample tested with buffer and sample tested with full length anti-DLL4. Full length anti-DLL4 contains all epitopes seen in anti-DLL4 F(ab′)_2_ except for the hinge neo-epitope which is formed by Fc cleavage and thus will deplete anti-CDR and anti-framework but not anti-hinge antibodies. Affinity-purified anti-CDR antibody was added at a concentration of 500 ng/mL to a panel of 14 serum samples from untreated cynomolgus monkeys. These sera were tested using different diluents to evaluate the effect of either no competition or competition with 50 *µ*g/mL of competitive molecule. Each sample was diluted either with assay diluent alone (no competition) or with diluent containing full length anti-DLL4 or with anti-DLL4 F(ab′)_2_. The diluted samples were then tested in the bridging ATA assay, resulting in average signals of 1.656, 1.388, and 0.260 absorbance units (AU), respectively. Thus competition with full length anti-DLL4 only reduced average signal by 16%.

The second assay format used a dual competition with anti-DLL4 F(ab′)_2_ and with Herceptin F(ab′)_2_ in the bridging assay. Anti-CDR antibodies are detected by calculating the change in signal between sample tested with anti-DLL4 F(ab′)_2_ and sample tested with Herceptin F(ab′)_2_. The same sample panel to which 500 ng/mL anti-CDR antibody had been added was competed with either Herceptin F(ab′)_2_, which will compete with anti-hinge but not anti-CDR ATA, or anti-DLL4 F(ab′)_2_, which competed with both anti-hinge and anti-CDR ATA. The average signals from the anti-DLL4 F(ab′)_2_ competition and the Herceptin F(ab′)_2_ competition were 0.250 and 0.653 AU, respectively, resulting in a 62% reduction in average signal using anti-DLL4 F(ab′)_2_ compared to Herceptin F(ab′)_2_.

Thus we were able to show clear signal drops in both competitive assays for 500 ng/mL anti-CDR antibody in the presence of a high preexisting ATA response, but the F(ab′)_2_ dual competition provided a more robust approach for the detection of anti-CDR antibodies in the presence of high levels of anti-hinge ATA. This differential competition method was therefore used to determine the presence or absence of anti-CDR ATA.

### 3.5. Final Method: ATAs to Whole Molecule

A titer method was used to evaluate ATA response to the entire anti-DLL4 F(ab′)_2_ molecule. Serum was diluted 1/20 and then titered in 1/5 dilution steps, eight dilutions in total. The titer cutpoint was set as 2 times the average signal of assay diluent wells. A similar method has been described [26].

Because every animal had a preexisting ATA (anti-hinge) response, including some animals with very high titers, the ability to detect a postdose titer change was problematic. Therefore, a method to set an individual cutpoint for each animal was also developed to determine if the ATA response to the whole molecule changed upon treatment. As shown earlier, preexisting ATA responses vary considerably between animals, but the responses over time within individual animals are more consistent. A pooled CV calculation method was used to compensate for this interanimal variability. This is based on the pooled variance method, which estimates the variance when the mean response may vary between animals, but where repeated samples from an animal are expected to have similar variability.

Two pretreatment samples separated by one week were obtained for each animal and titered in the assay. The standard deviation of the two pretreatment sample titers for each animal was calculated. The pooled standard deviation was calculated by averaging the individual prestudy standard deviations.

The range of titers that would be expected from normal variability was set for each animal using the pooled standard deviation by multiplying the pooled standard deviation by 2.33, the 99th percentile of the normal distribution, and then adding and subtracting this factor from each animal's average predose titer. Posttreatment sample titers that fell outside that range were thus considered to have been increased or decreased due to drug treatment.

### 3.6. Final Method: ATAs to CDR

Anti-CDR ATA responses were detected by testing each sample at a 1/20 dilution using the dual F(ab′)_2_ competition method. The sample drop score is the difference of the signals relative to the Herceptin F(ab′)_2_ signal. The drop score for each sample was computed as follows:

  
*H* = signal when 50 *μ*g/mL Herceptin F(ab′)_2_ is added to sample.
  
*D* = signal when 50 *μ*g/mL anti-DLL4 F(ab′)_2_ is added to sample.
  Drop score = [*H* − *D*]/*H* × 100%.

The cutpoint for anti-CDR positivity was set for each animal individually by adding 2.33 (the 99th percentile of the standard normal distribution) times the estimated standard deviation (SD) to each animal's baseline sample signal. The SD is estimated by pooling individual SDs across animals. The steps in computing and applying the anti-CDR cutpoint are the following:

- For each animal, two predose samples were taken and tested in the two competitive assays.
- For each animal, the individual mean as well as variance of the two pretreatment sample drop scores was calculated.
- The pooled SD was calculated by taking the square root of the average of the individual variances.
- Each animal's individual cutpoint was calculated by adding 2.33 times the pooled SD times to the individual mean.
- Any posttreatment sample that had a signal above its individual cutpoint was positive for anti-CDR.

Some animals had signals for one or both of the F(ab′)_2_ competitions that were above the accuracy limit of the spectrophotometer. For these samples, the drop score could not be computed and therefore the result was reported as indeterminate.

### 3.7. Detection, Titration, and Anti-CDR Assay Signal Changes in Samples

The titer and anti-CDR methods were used to analyze serum samples from cynomolgus monkeys that were dosed weekly with either vehicle (control group) or 5, 15, or 50 mg/kg anti-DLL4 F(ab′)_2_ [14], with five males and five females in each dose group. Nine doses in total per animal were given over eight weeks.

The changes seen in ATA titer for the study animals are summarized by dose group and by day in Table 1.

In the control group, 27 of 28 samples in the control group showed no significant titer change. One sample at day 29 showed a significant titer change; this may be a false positive due to the statistical basis of the method.

In the 5 mg/kg low dose group, the majority of samples showed an increase in titer posttreatment. The 50 mg/kg high dose group showed the opposite pattern, with most of the samples showing a decrease in titer. This may be due to drug interference in the ATA assay; however measured drug concentrations were below the drug interference level in most cases. This result could also be due to high dose tolerance [27, 28]. The 15 mg/kg mid dose group was split between decreasing, unchanged, and increasing titer.

### 3.8. Development of Anti-CDR ATA Could Be Detected in Some Animals Treated with Anti-DLL4 F(ab′)_2_

**Figure 5:**
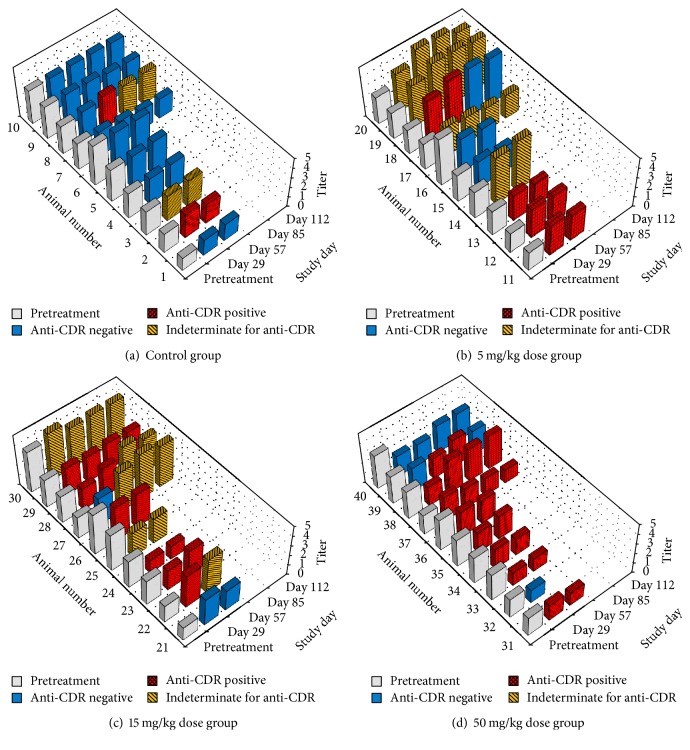
Response-time plots of ATA titer to the whole F(ab′)_2_ molecule and anti-CDR ATA positivity status by dose group. Solid light gray bar: pretreatment titer (average of two samples). Solid blue bar: posttreatment titer for anti-CDR negative sample. Horizontal hatched red: posttreatment titer for anti-CDR positive sample. Diagonal hatched yellow bar: posttreatment titer for anti-CDR indeterminate sample.

Anti-CDR antibodies were detected in all dose groups, as shown in Figures 5(a)–5(d). In the control group, three of the 28 posttreatment samples were anti-CDR positive, probably due to the statistical method of setting the cutpoints resulting in false positive outcomes. The cutpoint factor is chosen to give a predicted 5% false positive rate to ensure that more true positive samples are detected, but higher pretreatment rates may be seen in the actual study samples due to the small number of samples used to set the cutpoint, assay variation, or differences between samples used to set the cutpoint and the study samples. In the anti-DLL4 F(ab′)_2_ treatment groups, positive anti-CDR responses were seen in 18 of the 25 treated animals with interpretable results.

## 4. Conclusions

Accurate measurement and appropriate interpretation of data on the immunogenicity of a therapeutic protein are important to its successful development due to the potential impact of immunogenicity on safety and efficacy [2, 3]. During the development of F(ab′)_2_ therapeutic we encountered preexisting anti-F(ab′)_2_ antibodies at high titers in virtually every cynomolgus monkey serum tested.

These high preexisting ATA levels could potentially confound our ability to detect a drug induced ATA response (similar results were observed with a small set of human serum samples; data not shown). Here we report methods to assess both total and anti-CDR specific ATA reactivity in the presence of preexisting anti-F(ab′)_2_ antibodies to enable immunogenicity assessment. Cutpoints for each individual animal were used with both a titration method for total ATA reactivity and a dual competition method to detect anti-CDR specific ATA.

This dual approach enabled a more detailed assessment of the nature of treatment induced ATA responses, since ATA response both to the entire molecule and to the CDR could be evaluated, even in the presence of high titers of anti-hinge antibodies. These are informative for interpretation of toxicology data, since anti-CDR antibodies may neutralize the drug and thus reduce any on target effect; conversely, anti-drug antibody complexes with either anti-CDR or anti-framework antibodies may alter drug clearance or induce off-target toxicological effects via immune complexes.

In this investigation we used pretreatment samples to derive cutpoints for use as decision thresholds for individuals. Some apparently false positive anti-CDR antibodies were detected in the control animals, which we attribute to the statistical method for setting the cutpoint. This may also be due to variation in antibody titers over the course of the study unrelated to treatment, since the study duration was longer than our evaluation of antibody variation. An approach that could potentially mitigate this issue would be to use control animal samples taken over the entire course of the study to calculate the pooled standard deviation and thus account for this variability. This method could also be extended by defining cutpoints that support further sample dilution to reduce the number of indeterminate samples.

Future work may include the analysis of neutralizing activity of anti-CDR specific ATA and study of mechanism and significance of preexisting anti-hinge antibodies and application of this method to human samples and clinical trials. We and other authors [20, 22] have also observed preexisting anti-F(ab′)_2_ reactivity in human serum samples from untreated subjects. This approach should be applicable to human studies, where either multiple pretreatment samples, study control group samples, or a historical control from a study in a comparable population could potentially be used to establish the pooled standard deviation.

## Figures and Tables

**Table 1:** Changes in titer by study day and by dose group.

Group	Change in titer	Number of animals/total number of animals			
		Day 29	Day 57	Day 85	Day 112
Control	Decreased	0/10	0/10	0/4	0/4
	Unchanged	9/10	10/10	4/4	4/4
	Increased	1/10	0/10	0/4	0/4

5 mg/kg	Decreased	1/10	1/10	0/4	0/4
	Unchanged	2/10	2/10	1/4	1/4
	Increased	7/10	7/10	3/4	3/4

15 mg/kg	Decreased	5/10	3/10	0/4	0/4
	Unchanged	2/10	4/10	2/4	2/4
	Increased	3/10	3/10	2/4	2/4

50 mg/kg	Decreased	8/10	7/9	1/4	3/4
	Unchanged	1/10	1/9	2/4	0/4
	Increased	1/10	1/9	1/4	1/4
